# ADRENALINE, a Learning Game to Improve Prescribing Skills in Undergraduate Medical Students: Descriptive Study

**DOI:** 10.2196/66334

**Published:** 2025-09-11

**Authors:** Cecile Marie Yelnik, Aurélie Daumas, Yanele Poteaux, Annie Standaert, Natacha Grimbert, Raphaël Favory, Pierre Ravaux, Marc Lambert, Katia Quelennec

**Affiliations:** 1 CHU Lille, Département de Médecine Polyvalentes et de Post-Urgences, Univ. Lille, Hôpital Calmette, Bd du Professeur Jules Leclercq, Lille, 59000, France, 33 320445479; 2 U1167 -RID-AGE- Facteurs de risque et déterminants moléculaires des maladies liées au vieillissement, Institut Pasteur de Lille, Inserm, Lille, France; 3 Internal Medicine and Therapeutics Department, La Timone, Assistance Publique-Hôpitaux de Marseille (AP-HM), Aix-Marseille Univ, Marseille, France; 4 DAPI, Univ. Lille, Lille, France; 5 Inserm, CHU Lille, U1286 -INFINITE- Institute for Translational Research in Inflammation, Univ. Lille, Lille, France; 6 Département de Pharmacie Officinale, UFR3S - pharmacieLille, France; 7 CHU Lille, Département de médecine intensive et de réanimation, Univ. Lille, Lille, France

**Keywords:** serious game, game-based learning, therapeutic, medical prescription, medical education, learning games

## Abstract

**Background:**

Junior doctors often demonstrate insufficient prescribing skills, highlighting the need to enhance undergraduate medical training in this area. Serious games (SGs) have proven effective in teaching knowledge and skills across various medical specialties including surgery and emergency care. To our knowledge, no SG specifically dedicated to prescribing has been developed to date. Our objective was to develop a new educational program, based on a learning game designed to enhance medical students’ competencies in safe and effective prescribing.

**Objective:**

This study aimed to describe ADRENALINE, a learning game designed to promote safe and effective prescribing, and to report feedback from sixth-year undergraduate medical students at our medical school after the first year of its implementation in the therapeutics curriculum.

**Methods:**

This study implemented an interactive educational program based on Kolb experiential learning theory to enhance safe and effective prescribing skills among sixth-year medical students. The program followed 3 phases: a preliminary in-person lecture introducing the SG ADRENALINE, autonomous gameplay, and a final debriefing lecture. ADRENALINE, accessible via university platform Moodle (Andrews Lyons) on multiple devices, was developed using MOSAIC software (Katia Quelennec), a software program created to develop evolutive SG based on real-life professional situations, and includes 20 realistic clinical scenarios of varying difficulty, requiring students to make therapeutic decisions and receive immediate feedback. Players advance through levels based on performance, with ongoing support from professors via feedback and a dedicated forum. The program was integrated into the therapeutic curriculum of Lille University, and participation was voluntary. All 598 sixth-year students were invited to access the game via email and to participate in pre- and postintervention surveys assessing usage patterns, satisfaction, and learning outcomes.

**Results:**

Between November 2023 and March 2024, 272 sixth-year students accessed the ADRENALINE program. Of these, 201/272 (73.9%) students completed at least one scenario and obtained scores ranging from 16.5 to 100 out of 100. Pretest survey responses (n=99 answers) indicated that 92/99 (93%) students identified as gamers and believed that SGs could be relevant for their medical education. Posttest survey responses (n=50 answers) reflected a high level of satisfaction among participants. Most students reported that ADRENALINE helps them apply academic knowledge in real-world context, feel more confident with prescribing and managing adverse drug reactions, improve their prescribing skills, and better prepare for the national Objective Structured Clinical Examination.

**Conclusions:**

We developed a learning game focused on medical prescribing, designed to be easily shared with other French-speaking medical schools. Although only 201/598 (33.6%) students engaged with this initial version, 85% (42/50) of the feedback received was positive, indicating strong student interest and supporting the educational value of a game-based approach to enhance prescribing skills among undergraduate medical students.

## Introduction

Prescribing is a complex task that requires a broad range of medical knowledge, clinical skills, and professional attitudes, all applied within a dynamic health care system and social context. In a systematic literature review, Brinkman et al [1] addressed the question of which competencies medical students should acquire by graduation to prescribe safely and effectively. They identified over 40 prescribing competencies, despite a lack of a strong consensus among Clinical Pharmacology and Therapeutics (CPT) teachers. Their review also revealed that many students perceive limited learning opportunities related to rational prescribing during their medical training and feel inadequately prepared for their future prescribing responsibilities [1]. Prescribing errors are frequent and can result in serious adverse events. Junior doctors, who are often the primary prescribers in hospital settings, are particularly prone to such errors, with evidence suggesting a higher error rate compared to their senior colleagues [2-6]. Educational interventions and structured training have been shown to reduce prescribing errors [7]. However, a study on CPT education across Europe found that traditional teaching methods (eg, lectures and written examinations) still dominate and are associated with lower levels of prescribing competence than active, problem-based learning methods such as clinical simulations or real-life prescribing practice [8]. In response, there is a growing need to adapt CPT education to better prepare students for rational and safe prescribing.

Digital educational resources, including e-learning programs, podcasts, simulations, serious games (SGs), and virtual or augmented reality, offer several advantages over traditional methods. These include flexibility in time and location, integration of multimedia and interactive content, and opportunities for repeated exposure to key concepts. A systematic review suggested that such tools could be effectively used to enhance prescribing education [9]. The European Association for Clinical Pharmacology and Therapeutics conducted a cross-sectional survey across Europe on the use of digital tools in CPT education [10]. The findings showed that although digital resources are widely adopted, most are limited to knowledge acquisition, as they are predominantly e-learning platforms. SGs, defined as games designed for purposes beyond entertainment, such as education or training, represent a promising pedagogical tool. Educational SGs, often referred to as learning games, have the potential to foster cognitive skills, behavioral change, and practical competencies [11]. Over the past decade, SGs have gained traction in medical education, with studies reporting high levels of student motivation, engagement, and satisfaction [12,13]. For example, a study involving 108 third-year medical students evaluated an SG focused on pulmonary embolism and found that it significantly enhanced student engagement and learning [14]. Although several SGs have been developed for health care students, particularly in fields such as surgery, emergency care, and pharmacy, to our knowledge, no SG to date has been specifically designed to develop undergraduate medical students’ competencies in clinical prescribing.

The objective of this study was to design a learning game aimed at enhancing undergraduate medical students’ competencies in safe and effective prescribing. This paper provides a detailed description of the ADRENALINE SG and presents user feedback from the first cohort of sixth-year medical students who piloted its initial version.

## Methods

### General Presentation of the Program

This interactive program was designed by the Professors of Therapeutics team, who have a background as medical doctors, to address our main objective of improving prescribing education. Our program was developed based on Kolb experiential learning theory (1984), which is particularly well suited to the learning of medical prescribing. This process involves complex decision-making and the application of knowledge in varied clinical situations, making experiential learning an ideal framework. The theory outlines 4 sequential phases that structured our program: concrete experience, reflective observation, abstract conceptualization, and active experimentation. The program was implemented in 3 phases: first, an in-person lecture introducing the SG and its learning objectives; second, independent gameplay by the students, and finally, a second in-person lecture dedicated to debriefing, conducted by the 3 professors of therapeutic from our university.

During the game, students engage in concrete experience by actively participating in realistic prescribing scenarios. This immersive approach provides learners with additional opportunities to encounter diverse clinical cases beyond those typically experienced in real-life settings, which can be limited or occur randomly during clinical placements. Importantly, real-time debriefing is provided at each stage of the game, allowing students to immediately reflect on their decisions and receive feedback on their performance. Following gameplay, students further engage in reflective observation by reviewing their overall performance and decisions, facilitated through detailed feedback on correct and incorrect answers. The subsequent phase, abstract conceptualization, takes place during debriefing sessions where theoretical knowledge is connected with practical experience, and any misunderstandings are clarified. Finally, active experimentation occurs during the final debriefing with instructors, allowing students to consolidate their learning and prepare for future clinical practice. By combining experiential learning with guided instruction and real-time feedback, the game enhances students’ competence and confidence in medical prescribing, providing a structured yet flexible environment to develop critical skills in patient safety.

All 598 sixth-year undergraduate medical students registered at Lille University were invited to attend the nonmandatory in-person lectures, and all students (including those who did not attend the in-person lecture) received internet-based access to ADRENALINE via email invitation. Participation was based on volunteering and integrated into the therapeutic education of our faculty. At each phase of the program, all students received email invitations, regardless of whether they attended the in-person sessions or accessed the game.

### SG Characteristics

ADRENALINE was developed using the MOSAIC platform (Katia Quelennec), which is based on the ScenariChain Opale, ScenariChain Topaze, and JavaScript software. MOSAIC was specifically designed to create adaptive SG rooted in real-life professional situations. ADRENALINE was adapted from e-Caducée, a learning game initially developed by our faculty of pharmacy to support the professional training of undergraduate pharmacy students [15]. The development of ADRENALINE resulted from a collaborative effort between professors of therapeutics from the Universities of Lille and Marseille. The project was approved and supported by the French National College of Therapeutics (CNET). ADRENALINE is accessible via the university’s Moodle (Andrews Lyons) platform only, not through open access or commercial apps, and is compatible with multiple devices, including tablets, computers, and smartphones.

**Figure 1.**
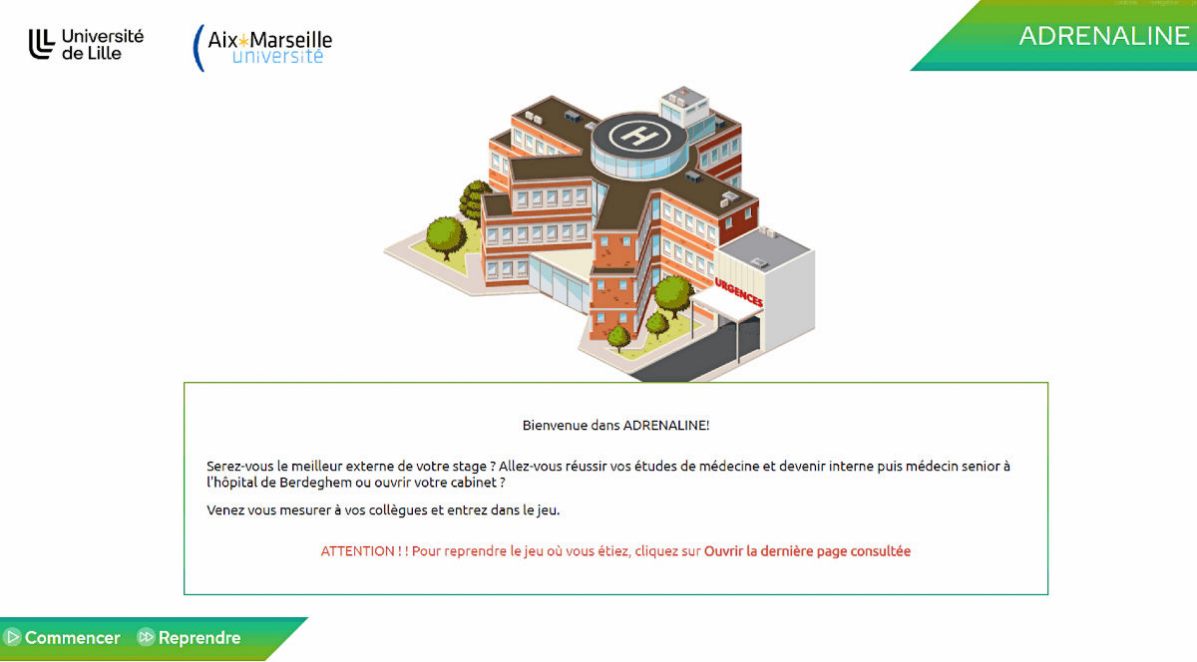
Home page of the ADRENALINE program. English translation of the French text: “Will you be the top medical student during your rotation? Will you succeed in your medical studies and become a resident, then a senior doctor at Berdeghem Hospital — or will you open your own private practice? Challenge your colleagues and jump into the game!”

ADRENALINE is a 2-dimensional, scenario-based SG designed to simulate common prescribing situations encountered in hospital settings. Players engage in interactive dialogues with game patients and health care professionals by actively selecting responses through a click-based interface. While the game does not allow free navigation within a simulated environment, players progress through clinical scenarios by making diagnostic and therapeutic decisions, ordering tests, and selecting appropriate prescriptions. In the game, the player assumes the role of a sixth-year undergraduate medical student at the fictional University Hospital of Berdeghem (Figure 1).

**Figure 2.**
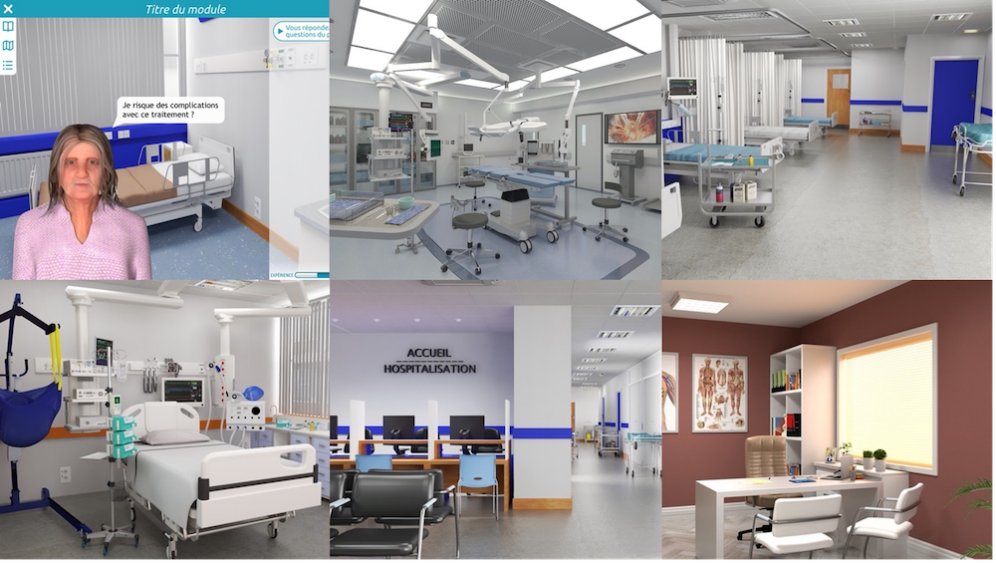
Examples of ADRENALINE’s graphical environment.

The gameplay revolves around realistic clinical situation scenarios where therapeutic decision-making and medical prescribing are central to the clinical problem. These scenarios were designed to reflect a wide range of clinical situations that medical students may encounter during their training, for example, answering questions from a supervising physician during hospital rounds, managing patients in the emergency department, or conducting consultations in an outpatient clinic (Figure 2).

The primary goal of the program is to progressively address all major therapeutic classes included in the official curriculum of the third cycle of medical education. This initial version of the game included 20 scenarios. Students should expect to spend approximately 2 hours to complete this initial version of ADRENALINE. Students respond to various types of questions, including multiple-choice questions, gap-fill exercises, and interactive activities such as identifying errors on prescriptions. The game comprises 3 levels of difficulty. As students progress, they gain increased responsibility in clinical decision-making, managing increasingly complex clinical situations. Their roles evolve from following patient visits to independently handling patient care, then taking emergency shifts, and finally conducting consultations alone directly with patients. A minimum of 60% correct answers is required to successfully complete the scenario, and students must successfully complete at least 80% of the scenarios to unlock the next level. Formative feedback from teachers, including links to additional resources when relevant, is provided throughout the game following each completed exercise. Players also had opportunities to provide open-ended feedback during the game by communicating with the Professors of Therapeutics via a pedagogical forum on the Moodle platform. They were additionally invited to provide open-ended feedback during the second in-person lecture, which was based on students’ questions and the most common errors. It provided professors with the opportunity to clarify medical reasoning in the context of iatrogenic events, explain the rationale behind specific prescriptions, and discuss practical prescribing procedures accompanied by patient counseling advice and real-life follow-up examples.

Prior to this study, students had acquired the necessary clinical knowledge and skills during their therapeutic courses at university.

### Evaluation Measurements

Students were invited to complete an anonymous internet-based pretest survey at the end of the first in-person lecture, as well as a posttest survey at the conclusion of the program, administered during the second in-person lecture. A reminder email containing a QR code linking to the surveys was sent to all sixth-year students, allowing those who did not attend the in-person lecture to complete the posttest survey if applicable. Pretest surveys included questions about students’ gaming habits and their intended use of ADRENALINE. The posttest survey was designed to assess their satisfaction after playing the game. Many questions asked students to indicate their level of agreement with statements using a 4-point Likert scale (ranging from “not at all” to “yes, completely”) or with yes or no responses. An open-ended question was also included at the end of the posttest survey to capture students’ limitations and suggestions for improving ADRENALINE.

### Data Management and Analysis

Data were collected anonymously through an internet-based questionnaire. A descriptive analysis of students’ responses to the pre- and posttest surveys, their usage patterns of ADRENALINE, and their game performance was conducted using Excel (Microsoft) software.

### Ethical Considerations

The study was conducted in accordance with the principles outlined in the Declaration of Helsinki. All data collected were fully anonymized, and all participants provided informed consent electronically for the use of their questionnaire data for research purposes. As this was an observational, noninterventional study using anonymized data, it did not require approval from an ethics review board according to French legislation, specifically in compliance with the Public Health Code [16], which exempts noninterventional studies using anonymous data from mandatory ethics committee review.

## Results

### Participants

Among the 598 sixth-year students enrolled at Lille University, 272 (45%) logged into the ADRENALINE program at least once and 201 (34%) completed at least one ADRENALINE scenario between November 2023 and March 2024. Among the players, 49/201 (24%) restarted the game at least once. Final score ranged from 16.25 to 100 and the mean score was 71/100 (SD 31.6). Ten (5%) students achieved a perfect score of 100.

### Results of Pretest Surveys

**Figure 3.**
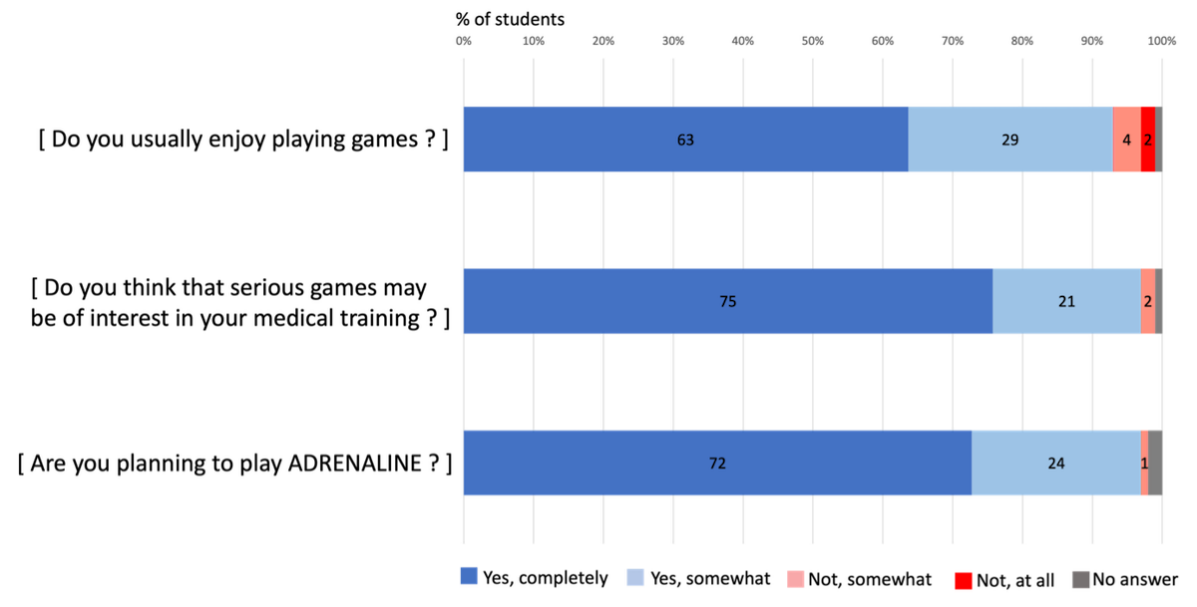
Pretest assessment of students’ interest in serious games.

Pretest surveys were collected from 99/598 (17%) sixth-year students. The majority, 92/99 students (93%), identified themselves as gamers and believed that SG could be relevant to their medical training (Figure 3).

Regarding usage pattern, 68/99 (69%) students planned to play alone, 25 (25%) planned to play either alone or in groups, and 5 (5%) planned to play exclusively in groups. Most students intended to use their personal computer to play ADRENALINE; approximately half also planned to use their smartphone, while only 3 (3%) students planned to use a university computer.

### Results of Posttest Surveys

Overall, 50/598 (8%) sixth-year students completed the posttest survey. Among them, 7 (14%) did not play to ADRENALINE. Reasons for not playing included lack of time (n=3), insufficient information about the game (n=1), intention to play later in preparation for the national Objective Structured Clinical Examinations (OSCEs) (n=2), and missing data (n=1). Consequently, responses were analyzed for 43 (15.8%) players.

From a technical perspective, students’ agreement regarding ease of use and the absence of technical issues was high. Except for 2 students, all played alone, mostly using their own computers. Twelve (24%) students played once, while the others played at least twice. Fifteen students played ADRENALINE more than 4 times.

**Figure 4.**
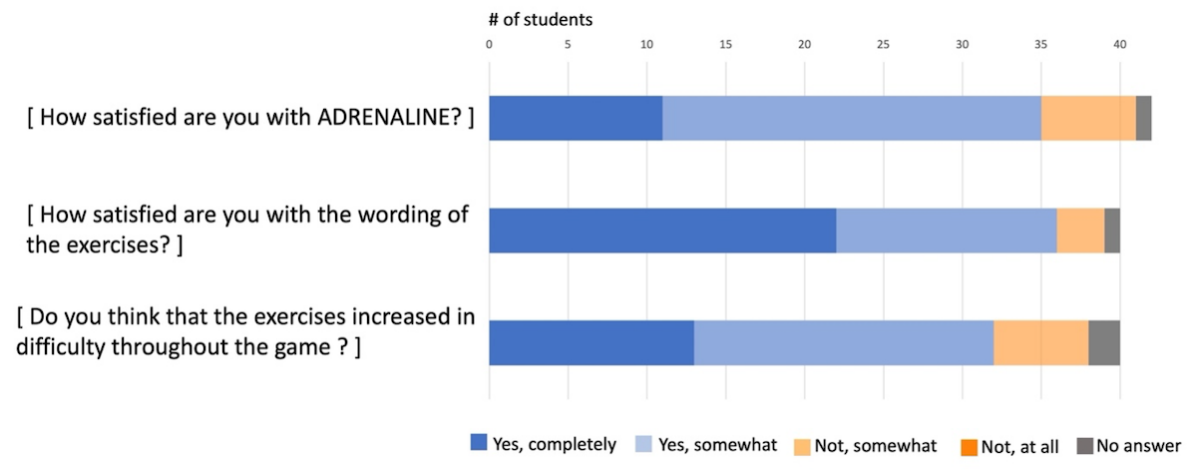
Posttest assessment of global students’ satisfaction.

Approximately 42/50 (85%) players enjoyed playing ADRENALINE and were satisfied with the wording of exercises and their level of difficulty (Figure 4).

**Figure 5.**
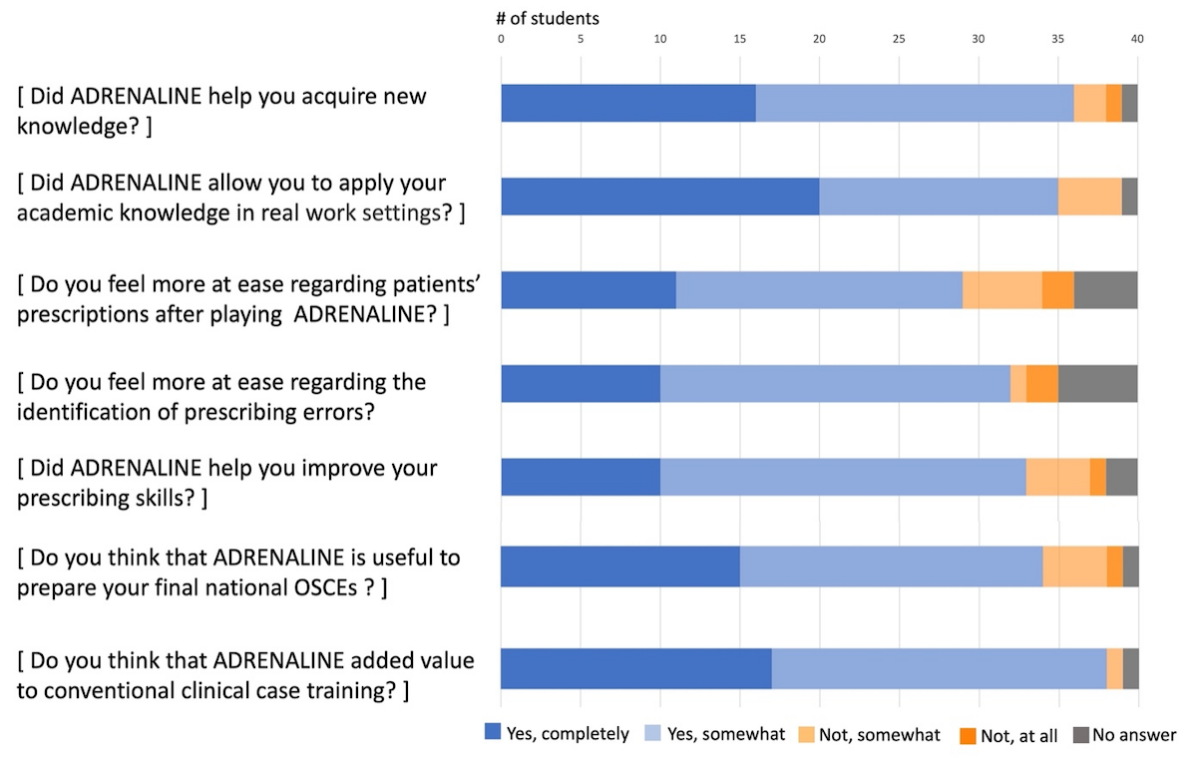
Posttest assessment of students’ opinion on ADRENALINE interest in their training.

The majority of players also believed that ADRENALINE helped improve their prescribing knowledge and skills (Figure 5). For 44/50 (88%) players, ADRENALINE facilitated the application of academic knowledge to real-life clinical settings. Compared to conventional clinical case training, 33/50 (65%) players considered the SG to add significant value for their medical education. Finally, 42/50 (85%) players found ADRENALINE useful for preparing for the national OSCEs.

## Discussion

### Principal Findings

Our study reported a high level of interest and satisfaction among sixth-year undergraduate medical students with ADRENALINE, an SG designed to enhance prescribing skills prior to their transition to the junior doctor role. Most students found the game enjoyable and perceived it as a valuable tool for improving both their knowledge and prescribing skills, offering added benefit compared to traditional lectures and clinical case–based teaching.

Two major trends are reshaping the traditional landscape of medical education: the decline in classroom attendance and the growing emphasis on competency-based learning that includes not only knowledge, but also skills and attitudes. A consistent global decline in undergraduate medical students’ attendance at lectures has been reported worldwide [17,18]. Technological advances in the digital and internet age have likely influenced medical students’ expectations for their education. The widespread availability of internet-based, on-demand educational resources may have contributed to a decreased interest in in-person lectures. One study examining the perspectives of medical students and faculty on the benefits of classroom attendance found that most students favored autonomy in choosing learning formats and viewed lecture recordings as acceptable substitutes for physical attendance [19]. In contrast, only 15% of faculty agreed with this view, instead emphasizing that attendance contributes positively to the development of professionalism. Another study involving first-year osteopathic medical students found no significant correlation between lecture attendance and academic performance, with students showing a preference for lecture capture and podcast as primary modes of content delivery [20]. However, these modalities may be insufficient for developing competencies beyond theoretical knowledge. Prescribing exemplifies this challenge; purely didactic instruction on pharmacology does not necessarily translate into safe and effective prescribing practice. The case-based learning method has been introduced to address this gap by contextualizing medical problems. Evidence from a randomized controlled trial (RCT) involving 80 postgraduate medical students indicated that case-based learning was more effective than traditional lectures in enhancing problem-solving skills [21]. Therefore, educational approaches that combine student autonomy with contextualized case-based methods are particularly needed for complex skills development such as prescribing.

Digital education methods and tools are increasingly being integrated into medical curricula and may help address both the decline in attendance and the need for skill-based learning by fostering autonomy and engagement. Game-based learning, in particular, has seen rapid growth over the past 2 decades [12,22,23]. A substantial majority of students report positive perceptions of SG in terms of engagement and learning [14,20]. The challenge-based structure of SG enables learners to actively participate in decision-making processes and receive immediate feedback, as exemplified by ADRENALINE. Nonetheless, existing meta-analyses have pointed to limitations in the evidence supporting the effectiveness of SG in medical education [24-27]. Methodological shortcomings include a limited number of RCTs, reliance on subjective evaluation metrics (eg, surveys and open-ended questions), and single-center study design. Authors have also noted heterogeneity among the SGs studied and the lack of direct comparisons between similar games in comparable settings. The most recent meta-analysis, which included only RCTs, found no significant differences between SGs and traditional educational methods in terms of knowledge acquisition, knowledge retention, cognitive or procedural skill development, or changes in attitude or behavior [27]. A minor improvement in self-reported confidence was the only consistent benefit associated with SGs. Thus, the added educational value of SGs remains to be firmly established. Our assessment of student satisfaction with ADRENALINE represents an initial step required in its implementation at our institution. The next step will involve evaluating its pedagogical impact by comparing student performance on national OSCEs according to their engagement with the game ADRENALINE. While we recognize that observational study design provides lower-quality evidence than RCTs, we believe conducting an RCT in this context may raise ethical concerns. Restricting access to ADRENALINE for some students could create inequality in preparation for a high-stakes national examination. Furthermore, RCTs may be subject to novelty bias, in which students’ enthusiasm for a new educational tool affects outcomes independently of its true pedagogical value.

For educators wishing to develop a similar SG in their own institutions, several key considerations emerged from our experience as developers and were echoed by the Professors of Therapeutics involved in the project. First, the content of clinical cases must be grounded in real-life medical practice to ensure authenticity and relevance. Designing scenarios that accurately reflect the types of prescribing decisions encountered by students and junior doctors was essential. This required close collaboration between content experts and pedagogical designers to strike the right balance between educational clarity and clinical realism. Second, the progression of difficulty within the game was a critical pedagogical choice. Based on our reflections and faculty feedback, we structured the game to move from relatively simple and sometimes exaggerated cases used to introduce basic principles toward more complex, nuanced clinical scenarios. This gradual increase in complexity supported learners’ engagement and confidence while fostering deeper integration of knowledge. Third, alignment with learners’ training level is essential. We specifically tailored the game to sixth-year medical students, ensuring consistency with the national curriculum and focusing on core pharmacological classes and prescribing competencies. We emphasized the importance of respecting curriculum standards while creating space for clinical reasoning and decision-making. From a development perspective, one of the main challenges was designing cases that were both didactic and clinically realistic, while remaining feasible within the format of a short interactive game. Another key learning was the value of integrating real-time feedback and debriefing after each scenario, which enhanced learner engagement and reflection. The involvement of experienced faculty members also helped ensure that the learning goals remained central throughout the design process. Finally, a major strength of this approach is its adaptability. From a perspective point of view, the game was designed to be transferable across institutions, allowing the platform to be enriched through the inclusion of a wider variety of clinical scenarios contributed by different faculties. This collaborative approach also supports the harmonization of therapeutics education across French medical schools. Furthermore, the national validation of the game’s content by the CNET ensures consistency with curricular standards and reinforces its pedagogical legitimacy. We believe these insights and practical considerations may assist educators in developing effective, scalable, and pedagogically robust SGs in medical education.

ADRENALINE has several limitations. First, only 201/598 (30%) sixth-year medical students at our institution participated in the game ADRENALINE during its first year of implementation, and the reasons for nonparticipation remain unknown. To improve uptake, we plan to send reminder emails highlighting the platform’s availability and its relevance for national OSCE preparation. The program will also be introduced during the university’s induction day. In addition, we aim to encourage student-led sharing of the game through informal networks and social media to increase awareness and engagement. Second, the high cost and time investment required to develop an SG may limit its widespread adoption. In the case of ADRENALINE, the licensing costs for the MOSAIC software were already covered by our institution, and its modular design allows for adaptation to various professional contexts. This enables potential implementation in other institutions without additional development cost, as the game has already been developed and tested at Marseille University. An agreement must be signed between Lille University and any institution requesting access to ADRENALINE for their students. We only ask instructors in therapeutics to contribute 5 new scenarios to enrich the game. This contrasts with the majority of previously published SGs (90.4%), which were limited to single-institution use. Third, as we tested a pilot version of the game, the number of available scenarios was limited. This restricts the range of prescribing situations represented, which may not fully reflect the diversity of real-world cases encountered by junior doctors. Therefore, the current version of ADRENALINE is not yet sufficient to evaluate its overall impact on students’ prescribing abilities. Finally, the types of exercises within the game are constrained by the capabilities of the MOSAIC software, which limits students’ ability to provide free-text or open-ended responses, unlike in real-life practice. Future developments, potentially incorporating artificial intelligence and chatbot technologies, could help address this limitation by enabling more naturalistic, flexible interaction.

ADRENALINE also has many strengths. First, the game integrated key factors known to enhance learning: attention, facilitated by the game’s visually engaging interface, active cognitive engagement supported by interactive, case-based real-life scenarios and a scoring system, feedback within the game from educators, and consolidation achieved through repetition of scenarios and a progressive increase in complexity. Second, ADRENALINE offers learners the opportunity to engage with a wide variety of realistic scenarios in a hospital setting, promoting contextualized learning. By playing as themselves, students are immersed in authentic clinical situations that closely mirror clinical reality. This experiential approach aligns with Kolb experiential learning theory, enhancing retention and transfer of skills to real practice. Finally, ADRENALINE is compatible with a wide range of digital devices and can be accessed anytime and anywhere, either individually or collaboratively. Its open accessibility allows for large-scale deployment across institutions, supporting wider dissemination and potentially contributing to national standardization of CPT education.

### Conclusion

In conclusion, despite a relatively low participation rate, with only 201/598 (33.6%) sixth-year students engaging with the game, a substantial proportion of users reported high levels of interest and satisfaction with the SG designed to improve prescribing skills. ADRENALINE appears to offer added educational value alongside conventional teaching methods in preparing students both for the national OSCE and for their future responsibilities as prescribers. Further research is now needed to assess the educational effectiveness of ADRENALINE, either as a standalone tool or in combination with other learning resources currently available within our institution.

## Abbreviations:

CNET: French National College of Therapeutics
CPT: Clinical Pharmacology and Therapeutics
OSCE: Objective Structured Clinical Examination
RCT: randomized controlled trials
SG: serious game
